# Split Femoral Nerve Due to Psoas Tertius Muscle: A Review with Other Cases of Variant Muscles Traversing the Femoral Nerve

**DOI:** 10.7759/cureus.1555

**Published:** 2017-08-09

**Authors:** Shehzad Khalid, Joe Iwanaga, Marios Loukas, R. Shane Tubbs

**Affiliations:** 1 Department of Anatomical Sciences, St. George's University School of Medicine, Grenada, West Indies; 2 Seattle Science Foundation; 3 Neurosurgery, Seattle Science Foundation

**Keywords:** femoral nerve, psoas muscle, psoas tertius, anatomy, variations

## Abstract

Leg pain from lumbar disc herniation is a common presentation. However, certain muscular and peripheral nerve variants may present similarly and represent an unrecognized etiology of femoral nerve dysfunction. Such cases might affect the outcome of specific treatment regimes. Therefore, recognition of these variations in anatomy may be useful to the clinician when treating the patient with medically refractory lower limb pain. Some reports have reported variant slips of the psoas and iliacus muscles, which may split the femoral nerve causing a potential risk for nerve entrapment. Herein, we report a very unusual variant of the psoas muscles, the psoas tertius, which pierced the femoral nerve into two parts. Additionally, the literature of other similar muscle variants is reviewed. Clinicians should be aware of anatomical muscular variants of the posterior abdominal wall and the propensity of such anomalies to result in distortion of regional neural structures. In this regard, the anatomy of the psoas tertius should be known.

## Introduction

The psoas major is a long fusiform muscle located on the side of the lumbar region of the vertebral column and the brim of the lesser pelvis. It joins the iliacus muscle to form the iliopsoas. As part of the iliopsoas, psoas major contributes largely to flexion in the hip joint. There is, however, a multitude of variations in this anatomical region, which the surgeons should be aware of, as they might commonly encounter these during their routine operative procedures. On the one hand, it is widely known that the femoral nerve is formed from the second to fourth lumbar vertebrae (L2 to L4) nerve roots at the level of the L4-L5 disc space and lies ventral to its posterior aspect in the majority of specimens studied. Surgeons should know that they are likely to encounter the trunk of the femoral nerve, not the nerve root contributions at the L4-L5 disc space. Direct trauma to the nerve can be avoided with proper technique, consisting of sequentially larger dilators, mobilization of neural structures without splitting them and avoidance of cutting instruments until full visualization of the disc space is achieved. On the other hand, documented variations of the iliopsoas muscle consist of 1) complete independence of iliacus and psoas major [1], 2) psoas major divided longitudinally into fascicles [2], 3) an accessory slip of psoas major lateral to the main muscle and separated from it by the femoral nerve [3], 4) a small detached portion of iliacus, the iliacus minor or iliocapsularis, arising from the anterior inferior iliac spine and inserted into the lower part of the intertrochanteric line of the femur, or into the iliofemoral ligament, and 5) a slip from iliacus may run medial to the psoas major. Herein, we report a very unusual variant of the psoas muscles, the psoas tertius, which pierces the femoral nerve into two parts. Informed consent was obtained for this study.

## Case presentation

**Figure 1:**
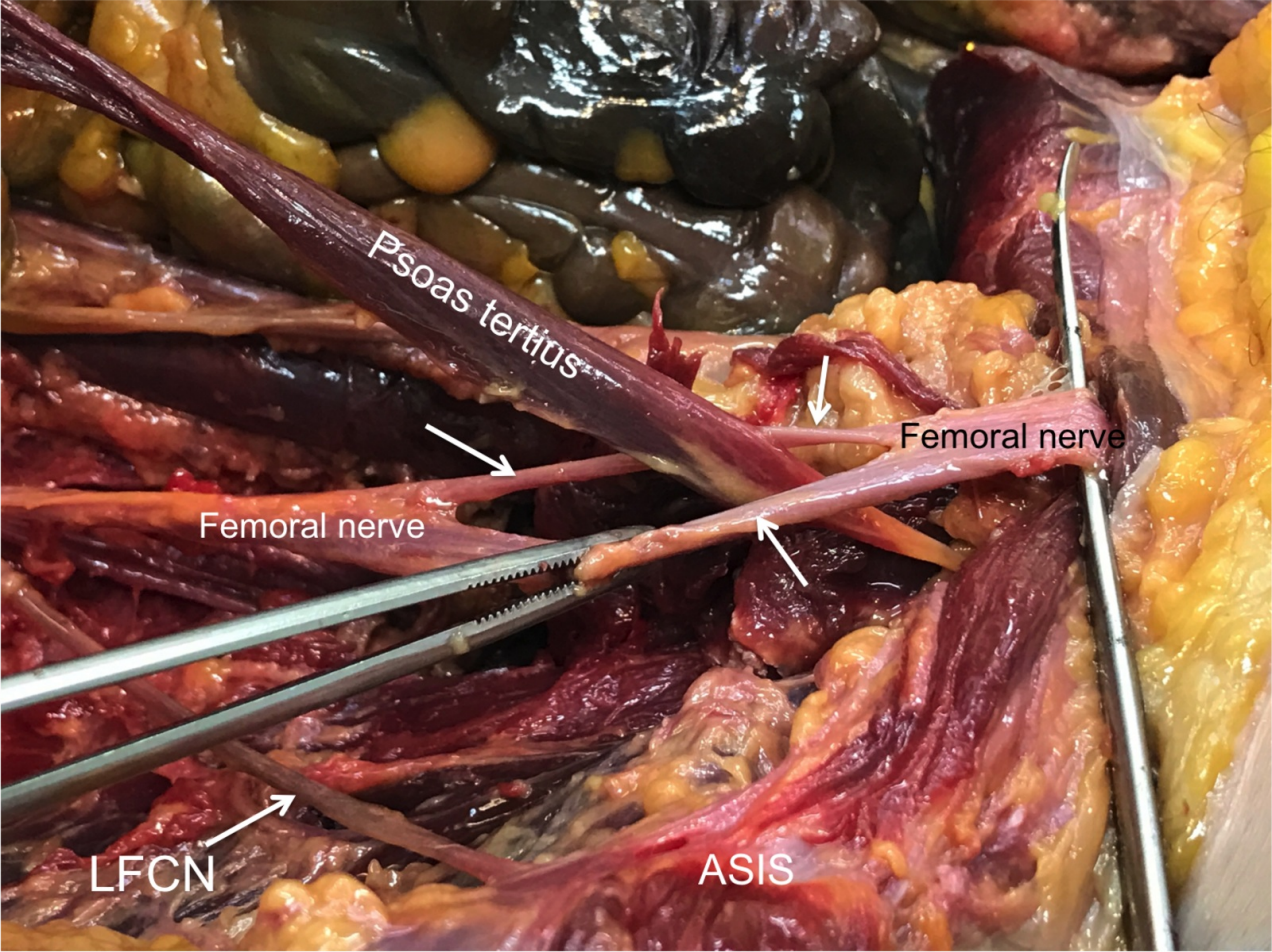
Right posterior abdominal wall of a 74-year-old female cadaver. The psoas tertius muscle has been disconnected from its origin and is shown traveling distally through the femoral nerve (white label indicates proximal nerve and the black label indicates distal nerve prior to traveling deep to the inguinal ligament) to fuse beyond this point with the tendon of iliopsoas. The perforation of the femoral nerve is shown with arrows. For reference, the right lateral femoral cutaneous nerve (LFCN) is shown traveling over iliacus toward the anterior superior iliac spine (ASIS).

During the standard dissection of the right posterior abdominal wall of an adult cadaver, an unusual muscle was identified. The specimen was that of a female cadaver who was 74 years old at death. The cause of death was pneumonia. The rare muscle in question was determined to be a psoas tertius muscle. It arose from the inner half of the 12th rib and from the tips of the transverse processes of the first four lumbar vertebrae. Distally, it pierced the femoral nerve (Figure 1) to fuse beyond this point with the tendon of the iliopsoas. The femoral nerve was pierced to form a roughly lateral two-thirds and medial one-third. The split occurred over 9 cm and then the femoral nerve parts reunited and traveled deep to the inguinal ligament in the normal fashion. There were no other anatomical variants, muscular or neural, noted on the left and right sides. No atrophy of the ipsilateral quadriceps femoris muscle was observed.

## Discussion

Clarkson, et al. (1889) discovered an unusual subdivision of the psoas muscle having seemingly four psoae muscles on each side during a standard dissection of a middle-aged male subject [3]. On the right side, the psoas magnus arose by mixed fleshly and tendinous slips from the sides of the bodies and anterior surfaces of the transverse processes of the upper four lumbar vertebrae, from the tendinous arches over the lumbar arteries and from the sides of all the intervertebral discs between the last thoracic and the fifth lumbar vertebrae. It also took origin from a slender tendinous arch that passed outward from the fascia at the level of the first lumbar intervertebral disc in the middle of the last rib. The psoas parvus muscle took origin by fleshy fibers from the side of the body of the first lumbar vertebra and upper part of the side of the body of the second lumbar vertebra and from the fibrous arch crossing the first lumbar artery. Its course was downward, in front of the psoas magnus, terminating in a long tendon, which was about three inches above the inguinal ligament, spread out into the iliac fascia, through which it was inserted onto the Ilio-pectineal line. From under surface of the tendon, at the point where it formed the above expansion, a few muscular fibers arose and formed a narrow sheet of muscle, which passed down to fuse with the internal surfaces of the iliacus and psoas major muscles. Interestingly, the psoas tertius arose from the inner half of the 12th rib, in intimate connection with the insertion of the quadratus lumborum and also from the tips of the transverse processes of the first four lumbar vertebrae between the origin of the psoas magnus and the insertion of the quadratus lumborum. It passed downward, in front of the quadratus lumborum and iliacus and ended in tendinous fibers, which fused near the level of the inguinal ligament with the tendons of the psoas magnus and psoas quartus. The case reported herein demonstrated a similar origin but during its course, pierced the femoral nerve at the end of the iliopsoas tendon (Figure 1). The psoas quartus was a small muscle, which arose by a small fleshy slip from the anterior surface of the lower and inner part of the tendon of the quadratus lumborum; also by a larger slip from the transverse process of the fifth lumbar vertebra and from the intertransverse ligament along its inner half. It passed downward to fuse with the tendons of the psoas magnus and psoas tertius at the level of the inguinal ligament. All the above muscles were separated by distinct cellular intervals, in which, ran branches of the lumbar and iliolumbar arteries for their supply. Branches of the femoral nerve were traced into each division of the psoas muscle. On the left side, the psoas magnus and psoas parvus had their origins, course, and insertions, similar to those on the right side; but there were no fibers arising from the tendon of the psoas parvus to correspond with those described on the right side. The psoas quartus arose by two slips from the transverse processes of the fourth and fifth lumbar vertebrae. The course and the insertion were on the right side.

A study performed by Spratt, et al. (1996) showed that variant slips of iliacus or psoas major were found unilaterally in four of the 68 cadavers [1]. A previously undocumented variant of iliacus was attached proximally to the lateral part of the iliolumbar ligament, ran inferiorly, posterior to the iliolumbar vessels and lay anterior to, and separate from, the iliacus. It traversed the femoral nerve 2 cm lateral to psoas major and 4 cm superior to the inguinal ligament, anterior to iliacus. One part of the tendon of this accessory slip attached distally to the lesser trochanter of the femur, the other continued as a long, thin tendon, which descended into the medial aspect of the thigh. Its distal attachment was not found. In another cadaver, a similar accessory slip of iliacus which arose more medially, from the ala of the sacrum, passed inferiorly, lateral to the femoral nerve, anterior to iliacus and joined the iliopsoas tendon to be inserted into the lesser trochanter. This variant did not, however, pierce the femoral nerve. In each of two cadavers, a lateral slip of psoas major traversed the femoral nerve 2-3 cm lateral to the main bulk of psoas major. It is definitely worth noting that psoas minor was present in all the specimens in which these variants were found.

In a study, Kirchmair, et al. (2001) dissected 63 lumbar plexuses from 32 cadavers to determine the topographical relation between lumbar plexus and psoas major muscle [4]. At the L4-L5 levels, variability in the course of the femoral, as well as the obturator nerve was then depicted. The lumbar plexus was situated within the psoas major muscle in 61 of 63 cases. In two of 63 cases, the entire plexus was confined posterior to the psoas major muscle. In 61 of 63 cases in which the lumbar plexus was situated within the psoas major muscle, the emergence of the individual nerves most often occurred in the posterior or postero-lateral surface. At the level of L4-L5, the femoral nerve has been shown to have a remarkable degree of branching, as well as the obturator nerve, were found within the psoas major muscle in the vast majority of the specimens.

Spratt, et al. found in bilateral dissections of 68 cadavers that four examples of them contained variant slips of iliacus and psoas major muscles unilaterally [1]. One of these variants was a previously undocumented accessory slip of iliacus, originating from the iliolumbar ligament, passing inferiorly anterior to iliacus and piercing the femoral nerve; its tendon split to be attached proximally to the lesser trochanter of the femur. Such anomalies might cause tension on the femoral nerve resulting in referred pain to the hip and knee joints and to the lumbar dermatomes L2, L3, and L4. The femoral nerve may pierce iliacus; enter the thigh more medially than usual between the femoral artery and vein; part of the nerve arising from L4 may run a separate course and leave the pelvis with the superior gluteal nerve to supply rectus femoris and vastus lateralis. The femoral nerve may also run buried in a cleft in the iliacus muscle, under psoas major.

Another study performed by Battaglia, et al. observed unilateral femoral and bilateral sciatic nerve variants in relation to the iliacus and piriformis muscle, respectively [5]. Further dissection of both the femoral nerve and accessory slip of the iliacus muscle was then performed to fully expose the anatomy. Multiple studies have reported variant slips of the psoas and iliacus muscles, which may split the femoral nerve causing a potential risk for nerve entrapment [6]. In a large study of 121 cadavers, Vasquez, et al. reported variations of iliacus and psoas muscles piercing the femoral nerve with piercing of the femoral nerve by a muscular slip, or a muscular slip/sheet covering the femoral nerve as it lay on the iliacus in 19 specimens (7.9%) [7]. Several entities exist which may cause femoral neuropathy and entrapment of the nerve is most likely to occur immediately distal to the inguinal ligament owing to the lack of anatomic protection [8]. No clinical finding is pathognomonic for femoral neuropathy. Similar findings of absent or diminished patellar reflex, quadriceps femoris muscle weakness or atrophy, weakness in hip flexion and sensory symptoms, such as pain in the iliac fossa, inguinal region, anterior thigh, and medial calf, may also suggest radiculopathy, plexopathy, or combined lesions of the femoral and obturator nerves [9].

## Conclusions

Variants in lumbar and lumbosacral plexus anatomy should be considered especially when a symptomatic lumbar disc herniation is refractory to medical treatment. Recognition of these anatomical variants, such as the psoas tertius, on imaging, might lead to earlier physiologic testing, better treatment outcomes and decreased patient morbidity.
